# The path to specialist multidisciplinary care in amyotrophic lateral sclerosis: A population- based study of consultations, interventions and costs

**DOI:** 10.1371/journal.pone.0179796

**Published:** 2017-06-22

**Authors:** Miriam Galvin, Padhraig Ryan, Sinead Maguire, Mark Heverin, Caoifa Madden, Alice Vajda, Charles Normand, Orla Hardiman

**Affiliations:** 1 Academic Unit of Neurology, Trinity Biomedical Sciences Institute, Trinity College Dublin, Ireland; 2 Department of Health Policy and Management, Trinity College Dublin, Ireland; 3 National ALS ClinicDepartment of Neurology, National Neuroscience Centre Beaumont Hospital, Dublin, Ireland; University of Toronto, CANADA

## Abstract

**Background:**

Amyotrophic Lateral Sclerosis (ALS) is a devastating neurological condition that requires coordinated, multidisciplinary clinical management. ALS is prone to misdiagnosis as its signs and symptoms may be non-specific, which may prolong patients’ journey to multidisciplinary ALS care.

**Methods:**

Using chart review and national register data, we have detailed the journey of a national cohort of ALS patients (n = 155) from the time of first symptom to presentation at a multidisciplinary clinic (MDC). Key milestones were analysed, including frequency of consultations, clinical interventions, and associated economic cost.

**Results:**

A majority of patients was male (60%), 65 years of age and over (54%), and had spinal onset ALS (72%). Time from onset of first symptoms to ALS diagnosis was a mean of 15.1 months (median, 11). There was a mean interval of 17.4 months (median 12.5) from first symptoms to arrival at the MDC, and a mean of 4.09 (median, 4) consultations with health care professionals. Electromyography and nerve conduction studies were among the most common interventions. Direct referral by a general practitioner (GP) to a neurologist was associated with reduced cost, but not reduced diagnostic delay. Bulbar ALS was associated with shorter time from symptom onset to diagnosis. Neurologist consultation in the first three consultations was associated with lower costs prior to the ALS clinic attendance but not a shorter time from first symptom to final diagnosis. Mean cost prior to attending the MDC was €3,486 per patient.

**Conclusions:**

Expedited referral to the multidisciplinary ALS clinic would have reduced costs by an estimated €2,072 per patient. Development of a standardised pathway with early referral to neurology of patients with suspected symptoms of ALS could limit unnecessary interventions and reduce cost of care.

## Introduction

Amyotrophic Lateral Sclerosis (ALS) is a progressive motor system degeneration with death from respiratory failure within three years of first symptom in 70% of patients [1].

There is increasing awareness of the overlap between ALS and Frontotemporal Dementia (FTD), and up to 50% of ALS patients exhibit cognitive and behavioural impairment [2]. There are currently no effective disease modifying therapies for ALS and management is symptomatic.

A diagnosis of ALS is primarily based on the physician’s interpretation of clinical symptoms and signs, and investigations to exclude other causes [3]. The wide range of presentations of ALS and the rapid clinical trajectory require a flexible approach to clinical care that is best provided in an integrated multidisciplinary setting [4].

Diagnostic delays are associated with clinical complexity, the patient either not recognising or denying early or intermittent symptoms, ineffective referral pathways, with patients not being referred to specialist physicians or being referred to a specialist other than a neurologist/ALS specialist, and the relative rarity and the consequent lack of familiarity with the condition [3, 5]. Earlier diagnosis can enable more effective symptom management care planning [6], and can alleviate caregiver burden.

Delays of over 12 months from the time of first symptoms to diagnosis of ALS have been reported [1]. Mitchell et al. [7] showed that diagnostic timelines have remained consistent over a 20 year period (1998–2008), time from first symptom to diagnosis is a median 12 months, and that delays were associated with clinical complexity and delays in referral both within primary and secondary care services.

Symptoms of ALS may raise many potential differential diagnoses. Disease onset is generally classified as being bulbar, spinal or respiratory, although a proportion of patients present with cognitive or behavioural impairment. Assessment by a neurologist at the first or second consultation has been associated with a shorter time to ALS diagnosis [7]. Delays in diagnosis can account for a significant proportion of total illness duration, and represents a missed opportunity to commence to manage patients’ symptoms. Diagnostic delay may also prohibit many patients from enrolling in clinical trials at an early stage of disease, when the likelihood of benefiting from experimental treatments might be greater [8]. Diagnostic delay also leads to delays in referral to multidisciplinary clinics (MDCs), which can improve patient outcomes [9].

In Ireland, a national referral centre for ALS care in Dublin provides multidisciplinary care with physiotherapists, occupational therapists, neuropsychologists, speech and language therapists and nutritionists. Previously, in a sample of 35 newly diagnosed ALS patients, we found that there was a mean interval of 19 months (median 14.6) from first symptoms to first review at the MDC and that patients had an average of four contacts with health care professionals and 4.8 investigations/tests prior to their first MDC visit [10]. Here we explore the journey of 155 ALS patients from symptom onset to first engagement with health services, and initial visit to the national specialist ALS MDC, and identify the factors that contribute to delays in accessing multidisciplinary care. We also assess the health economic consequences of both diagnostic delay, and delays in referral to speciality services, and explore the factors that may reduce delay and enhance diagnostic efficiency.

## Methods

155 patients attending the National ALS Clinic, and fulfilling the El Escorial diagnostic criteria for Possible, Probable or Definite ALS participated in a cohort study. Details of the journey from first symptoms to MDC attendance at Beaumont Hospital were obtained by contemporaneous chart review, which included a semi-structured interview performed by one of the authors (OH) at the time of the first clinic visit. Administrative and demographic information was recorded and the patient journey was documented under a number of headings. These included: first symptom, date of first symptom, date of diagnosis, El Escorial Criteria at the time of presentation at the specialist clinic, site of onset, date of first visit to the physician regarding symptoms, subsequent referrals and referral dates, investigations carried out during the referral phase, alternative diagnosis, and date of ALS diagnosis. Patients’ status regarding public health coverage or private health insurance was noted. Data were extracted from the national ALS Register where possible, and the remaining data were sourced from the medical chart review. Ethical approval was received from Beaumont Hospital Medical Research Ethics Committee review board (REC REF 12/84). Patient clinical details were available through the National ALS Register, for which they had consented to inclusion of their codified clinical and demographic data.

Consultations, diagnostic tests, clinical interventions and hospitalizations were quantified. The time from first symptom to key milestones in the patients’ journey to the ALS clinic was analysed. The milestones include GP consultations, referral to other health care professionals (non-neurologists), referral to a neurologist, attendance at the ALS clinic, and diagnosis. The economic cost of care was quantified from the perspective of the public sector provider of services, and the potential cost savings were estimated from improved referral to the clinic.

### Statistical analysis

Regression analyses were performed including the following variables: time from symptom onset to diagnosis (in months) as a dependent variable, and specified the following predictors: age, private health insurance status, gender, distance from the multidisciplinary clinic, bulbar or spinal symptom onset, and a binary variable of whether a patient moved to Ireland within the last three years. Further linear regression models included the same dependent variable, and the following predictors: age, private health insurance status, gender, bulbar or spinal symptom onset, diagnostic certainty at time of presentation to specialist clinic (El Escorial criteria), stage of ALS (Kings and MITOS staging systems [11, 12]. A third model then added a binary variable indicating patient attendance at a neurologist in the first three consultations.

Tobit regressions used the following dependent variables, 1: total healthcare spending from first symptom to diagnosis; 2: spending on consultations; 3: spending on procedures and hospitalizations. The predictors were the same as the three linear regressions models.

A logistic regression examined the predictors of referral to a neurologist in the first three consultations; the independent variables were age, gender, private insurance enrolment, site of onset.

An association between the presence of an alternative diagnosis and three variables: surgery, costs, and site of ALS onset was quantified. The associations between referral to a neurologist in the first three consultations and three variables: length of time from first symptom to diagnosis, costs, and site of ALS onset; and between costs and the time from first symptom until diagnosis were also quantified. T-tests were used to examine the relationship between continuous and binary variable, chi-squared tests examined the relationship between categorical variables, and the correlation coefficient examined the relationship between continuous variables.

## Results

A majority of patients was male (60%), 65 years of age and over (54%), and had spinal onset ALS (72%). 12 of 155 (7%) patients were noted to have evidence of severe cognitive impairment consistent with a frontotemporal dementia at the time of diagnosis. In all cases, this finding had not been identified prior to definitive diagnosis of ALS in the Specialist Clinic.

In total, 54% of all patients had private health insurance. Table 1 shows the characteristics of patients.

**Table 1:** Patient characteristics—Summary statistics.

	Number (percentage)
**Gender**	
Female	62 (40%)
Male	93 (60%)
**Age group**	
< 45	7 (5%)
45–54	21 (14%)
55–64	44 (29%)
65–74	57 (37%)
75+	26 (17%)
**Site of onset**	
Bulbar	43 (28%)
Spinal	112 (72%)
**El Escorial Criteria**	
Definite	72 (49%)
Probable	44 (30%)
Possible	31 (21%)
**Alternative diagnosis**	20 (13%)
**Insurance**	
Public	62 (40%)
Private	83 (54%)
Public but some private services	4 (3%)
Unknown	6 (4%)

Mean age was virtually identical for privately insured and uninsured patients (64.5 years). Of female patients 59% were privately insured, compared to 51% of males. Twenty patients (12%) received at least one alternative diagnosis prior to ALS diagnosis, and one patient received two alternative diagnoses. Of these, twelve patients had one structural misdiagnosis, and one patient had two structural misdiagnoses. Structural misdiagnoses included cerebrovascular disease, hiatus hernia with reflux, cervical myeloradiculopathy, and lumbar radiculopathy. Two patients had an immunological misdiagnosis (myasthenia gravis, transverse myelitis), and five patients had a degenerative misdiagnosis (neuropathy, arteriopathy, myopathy). In fourteen cases the alternative diagnosis was made by a GP, in two cases by a chiropractor, while a neurologist, neurophysiologist, rheumatologist, cardiologist, surgeon, and hospital physician each made one alternative diagnosis. In total, 11% of patients with bulbar onset ALS had one or more alternative diagnoses, compared to 15% of patients with spinal onset ALS.

### Consultations

Patients had a mean of 4.09 consultations with health care professionals before arrival at the MDC (median, 4), most commonly with general practitioners and neurologists. The first recorded consultation was with a GP for 141 patients (91% of cases), and the remaining patients reported that their first consultation was with a physiotherapist, a hospital-based consultation, or general hospital based physician. The first referral from the GP was to a neurologist in 44 patients (28%), whilst GPs also commonly made a first referral to ear, nose and throat specialists (ENT) (13 patients), orthopaedic physicians (12), and physiotherapy (7).

97 patients (62%) attended a neurologist at least once during the first three consultations. The remaining 38% of patients most commonly attended an orthopaedic physician (N = 11), but also attended a range of other specialties including rheumatology, ENT, cardiology and medicine for the elderly.

A logistic regression model examined the following predictors of referral to a neurologist in the first three consultations: age, gender, private insurance enrolment, bulbar or spinal onset. Age was the only significant predictor at a standard confidence threshold, and had an odds ratio of 0.97 (P = 0.047) for attendance at a neurologist. Patients who were not referred to a neurologist in the first three consultations were likely to have been referred to ENT specialists, orthopaedic physicians, and physiotherapy.

There was no significant difference between bulbar and spinal onset patients in terms of the likelihood of being referred from a GP directly to a neurologist (P = 0.96), nor in the likelihood of attending a neurologist in the first three consultations (P = 0.24).

Table 2 shows the number of referrals to health care professionals, attendances at emergency departments, and the number of hospitalisations for the 155 patients.

**Table 2:** Clinical referrals.

	Total	Mean	No. patients (%)	Minimum	Maximum
**GP review**	156	1.01	155 (100%)	1	2
**Diagnostic Admission to Hospital**					
Emergency Admission	6	0.04	6 (4%)	0	1
Scheduled Admission	41	0.26	31 (20%)	0	4
**Clinical Opinion Review: Physician**					
Neurology	148	0.95	125 (80%)	0	3
Neurology/Neurophysiology	158	1.02	155 (100%)	1	2
Rheumatology	12	0.08	10 (6%)	0	2
General Medicine	12	0.08	12 (8%)	0	1
Cardiology	4	0.03	3 (2%)	0	1
Old Age Medicine	4	0.03	4 (3%)	0	1
GI physician	3	0.02	2 (1%)	0	1
Respiratory Physician	2	0.01	2 (1%)	0	1
**Clinical Opinion Review: Surgeon**					
Orthopaedic surgery	18	0.12	16 (10%)	0	2
Otorhinolaryngology	15	0.10	14 (9%)	0	2
General Surgery	11	0.07	9 (6%)	0	2
Neurosurgery	7	0.05	5 (3%)	0	2
**Clinical Opinion Non Physician/Surgeon**					
Physiotherapy	11	0.07	10 (6%)	0	1
Chiropractor	7	0.05	6 (4%)	0	1
**Other Review** ^**a**^					
Other	19	0.12	18 (12%)	0	2
Total	634	4.09	-	2	8

^a^ Other includes Occupational Health Physician, Dental Surgeon, Occupational therapist, Neuropathologist, Electrophysiologist, General radiologist, Infectious Diseases Physician, Psychiatrist, Pain specialist, Memory clinic, Haematology Physician, Osteopath, Rapid access stroke prevention clinic, Rehabilitation Medicine, Neuropsychologist, Unidentified

### Tests and interventions

All patients eventually diagnosed with ALS underwent routine diagnostic investigations including hematologic and biochemical testing, thyroid function studies, lumbar puncture, MRI of the brain and spinal cord, and neurophysiologic studies (EMG and nerve conduction studies).

Forty six (29%) patients were admitted to hospital for diagnostic work up, and of these 13% were admitted through the emergency department. Additional tests performed included chest x-ray (9), muscle biopsy (7), celiac screening (1); barium swallow (1), laryngoscopy and endoscopy (4).

Patients with an alternative diagnosis were more likely to undergo surgery (2-sided T-test; P = 0.006). Of the patients who underwent surgery before attending the MDC, four had an alternative misdiagnosis of lumbar spondylosis, and one patient underwent a second surgery for the same symptoms prior to the correct diagnosis of ALS. Two patients had a diagnosis of carpal tunnel syndrome and underwent surgery for median nerve entrapment. All were subsequently diagnosed with spinal onset ALS, and the symptoms that led to their surgery could later be attributed to ALS.

### Time from first symptom to final diagnosis

Table 3 shows the mean and median time (months) from onset of first symptoms to key junctures in the patient journey. The table is organised into the following key milestones: first consultation with a general practitioner (“GP”), referral from a GP to a specialist non-neurologist (“Referral”), referral to a neurologist (“Neurologist”), ALS diagnosis (“Diagnosis”) and first attendance at the multidisciplinary ALS clinic (“Clinic”). In each case the data are right skewed as the mean exceeds the median.

**Table 3:** Temporal pathway: Time from first symptom (months).

All patients	GP	Referral	Neurologist	Clinic	Diagnosis
Median	3	6	8	12.5	11
Mean	5.2	8.6	11.3	17.4	15.1

Time from onset of first symptoms to ALS diagnosis was a mean of 15.1 months (median, 11). There was a mean interval of 17.4 months (median 12.5) from first symptoms to arrival at the MDC. The mean time from symptom onset to contact with a general practitioner was 5.2 months (median, 3).

Referral to a neurologist in the first three consultations was not significantly associated with the length of time from first symptom to diagnosis.

Linear regression found that time to diagnosis was four months shorter for bulbar onset patients compared to spinal onset patients. No other predictors had a significant effect (Table A1 in S1 Appendix). When the Kings Staging score was included as a predictor and the model restricted to patients staged within 100 days of diagnosis, the Kings Stage [11] was the only significant predictor (P = 0.04, coefficient = 2.25).

For the patients who attended a neurologist during the first three consultations after symptom onset, there was no significant difference in time to diagnosis compared to other patients (14.7 months versus 15.4 months). Patients who received a misdiagnosis had a non-significant increase in time to diagnosis (16.1 versus 14.7 months).

### Cost

Table 4 shows the cost of diagnosis from the perspective of health care providers. Based on international recommendations (AAN and EFNS) [13,3] the cost of a standardised work up for diagnosis of ALS (including GP and specialist consultation, routine haematology and biochemistry, neuroimaging, lumbar puncture and neurophysiology) is €1,414 if performed on an outpatient basis. If performed with an overnight stay in a hospital this may increase cost by a mean of €426 per night, based on national DRG unit costs for neurology-related admissions. For an elective diagnostic workup with a length of stay of five days, the cost is €3,544. Admission through the ED further increases cost by €271.

**Table 4:** Cost estimates.

	Unit cost	Total cost	Mean cost
	€	€	€
**Consultations**			
GP	50.00	7,750	50
ED	270.99	1,626	10
Hospital consultation	131.45	5,390	35
Neurosurgery	131.45	920	6
Rheumatology	131.45	1,577	10
Neurophysiology	131.45	20,506	132
ENT	131.45	1,972	13
Neurology	131.45	20,375	131
Surgical	131.45	1,183	8
Physiotherapy	60.00	660	4
Orthopaedic	131.45	2,498	16
GI physician	131.45	394	3
Chiropractor	60.00	420	3
Radiologist	131.45	526	3
Cardiology	131.45	526	3
Consultant physician	131.45	1,577	10
Pulmonary specialist	131.45	263	2
Geriatrician	131.45	526	3
Other	60.00	1,140	7
Subtotal: consultations		69,829	451
**Tests and procedures**			
Radiology	720.00	111,600	720
Hospital admission	4,631.15	189,877	1,225
Surgery	9,504.00	66,528	429
Neurophysiology (EMG, NCS)	250.00	41,250	266
Treatment	300.00	6,000	39
Bloods	100.00	15,500	100
Lumbar puncture	163.00	25,265	163
Specific tests	50.00	1,000	6
Other	150.00	13,500	87
Subtotal: interventions		470,520	3,036
Total		540,349	3,486

The mean additional cost for a patient journey that deviated from the guidelines was €2,072, based on a guideline cost of €1,414 and the actual mean cost of €3,486. Surgical intervention related to ALS symptoms, which was performed on seven occasions, increased the cost of the pre-diagnostic journey by a mean of €9,504 per surgery (mean of €426 per patient).

In general, attending a neurologist in the first three consultations after symptom onset was inversely associated with costs (€2,716, versus €4,777 unpaired T-test, P < 0.001). The primary source of cost savings was hospitalisations without surgery (rates of 0.14 versus 0.41, P = 0.004), whereas there were no significant differences in spending on surgical intervention, radiology, lumbar puncture, and investigative tests such as electromyography and nerve conduction studies. When a dummy variable was added to the regression to indicate whether a patient attended a neurologist in the first three consultations, this was not significantly predictive of the time from first symptom to diagnosis, but it was a significant negative predictor of total spending, spending on interventions, and spending on consultations. Neurological review at the time of first or second consultation was also inversely associated with costs (€2,336 versus €3,997, unpaired T-test, P = 0.01). This was a function of enhanced diagnostic accuracy leading to significant reductions in radiology utilisation and hospitalisation without surgery. (Regression models are shown in Tables A2 and A3 in S1 Appendix).

There was a weak positive correlation between costs and the time from first symptom until diagnosis (R = 0.15).

Regression analysis found no significant difference in terms of healthcare costs or time to diagnosis by insurance status.

Misdiagnosis led to an increase in cost. This was significantly higher for patients who underwent surgical intervention, which is unsurprising as surgery is the single largest unit cost in Table 4.

## Discussion

### Time

Patients with ALS experience significant delay in achieving a definitive diagnosis. Our study confirms this finding. While the majority of ALS patients attended their GP as the first point of engagement with health services, there was a mean delay of five months from first symptom to first presentation- usually to a general practitioner. Further delays ensued with a mean of 17 months from symptom onset to attendance at a multidisciplinary clinic. Spinal site of onset was associated with a four month increase in diagnostic delay.

This finding confirms our earlier study which noted a mean interval of 19 months (median 14.6) from first symptoms to arrival at a MDC, an average of four contacts with health care professionals, and 4.8 investigations/tests prior to their first MDC visit [10].

Our data also demonstrate a significant interval from first symptom to time to diagnosis among ALS patients (mean, 15.1 months; median, 11 months), and this time lag is positively associated with cost. Unnecessary costs may arise from consultations, diagnostic tests, and unnecessary surgical interventions. Accordingly, there may be a need to accelerate access to multidisciplinary ALS care, and for more rapid diagnosis of ALS to improve quality of life and prevent unwarranted spending. [14]

ALS stage at time of presentation to the multidisciplinary clinic, using the Kings but not the MITOS staging system, was a predictor of time from first symptom to diagnosis [11, 12]. While a proportion of those with later stage of disease are likely to represent rapid progression, it is also possible that a subcohort of patients with more slowly progressive disease may not have been made aware of the availability of a MD clinic, or were reluctant to attend. The former category may have a more rapid (and less costly) journey to the clinic, whilst the latter may have had a slower (and more costly) journey. The study was not powered to detect these subgroup differences.

This analysis did not find evidence that recent residence in another country impacted these outcomes, or that distance from the MDC was an important factor.

A prolonged diagnostic process is likely to have a negative impact on the psychological well-being of patients and their families and result in a period of stressful uncertainty and anxiety [8]. Diagnostic delay also leads to delays in referral to multidisciplinary clinics, which can improve patient outcomes [9]. International recommendations suggest that multidisciplinary care should be available for people affected by ALS; attendance at multidisciplinary clinics extends survival [9], enhances quality of life [3, 15] reduces emergency hospital admissions [16] enhances access to clinical professionals [17] and appears to offer good value for money [18].

### Referrals

Following presentation to a GP, it is perhaps of concern that only 28% of patients were referred directly to a neurologist, despite all having neurological symptoms. Patients did not significantly differ by private insurance status in terms of time from symptom until diagnosis or costs of care.

Age, the only significant predictor at a standard confidence threshold, had an odds ratio of 0.97 (P = 0.047) for attendance at a neurologist in the first three consultations. This may reflect a slight increase in likelihood of referring older patients to non-neurologists. There were no significant predictors for attendance in the first two consultations. The remaining patients were more likely to be referred to ENT specialists, orthopaedic physicians, and physiotherapy. This may indicate that the clinical judgement and referral patterns of primary care physicians were based primarily on the anatomic site of presenting symptoms, rather than an analysis of a differential diagnosis that might include ALS. This is not surprising as most general practitioners are unlikely to encounter more than one or two patients with ALS throughout their careers.

Educational tools such as the “red flag” systems for general practitioners [19] are of value in this regard. Previous studies have indicated that the presence of a “red flag” symptom warrants prompt referral to a neurologist [20]. Review of our data revealed that the first symptom experienced by almost 70% (114) patients represented a “red flag” symptom that should have triggered a differential diagnosis of ALS. Such symptoms include progressive changes in speech and swallowing, progressive gait disturbance, painless change in hand function, head drop, and foot drop. However, the presence of a “red flag” symptom was not significantly correlated with time from first symptom to diagnosis, cost, or the likelihood of attending a neurologist in the first two or three consultations.

Our findings that misdiagnosis did not significantly impede the diagnosis of ALS differs from previous studies which have shown that misdiagnosis can delay the definitive diagnosis of ALS by approximately 9–13 months [7]. Cellura et al. [21] found approx one third (31.1%) of patients received another diagnosis before ALS, and this was associated with a lengthier diagnostic delay (median 15 months), relative to other patients (median 9 months) (p < 0.001), and Paganoni et al. [8] reported that 27–61% of ALS patients receive an alternate diagnosis prior to ALS.

Assessment by a neurologist at the first or second consultation has been associated with a shorter time to ALS diagnosis [7]. By contrast, in our study, attendance at a neurologist within the first three visits was associated with lower costs but not a shorter time from first symptom to diagnosis. Although not evidence based, we posit that neurologists are more likely to consider ALS as part of the initial differential diagnosis, and accordingly take less costly approached towards clinical investigation than would non-specialists. However, in Ireland there remains significant waiting lists for neurological appointments, and this may explain the absence of a significant relationship between neurological consultation and time to definitive diagnosis.

### Scheduled and non-scheduled admissions

Six patients attended the emergency room, and 41 were admitted to hospital for elective diagnostic work. While in some cases, admission was based on an erroneous diagnosis of cerebrovascular accident, patients also presented to the emergency room with longer standing neurological symptoms. None of these patients had been reviewed by a neurologist prior to presentation. For those electively admitted, it was not possible to establish whether the admission was under the direct care of a neurologist, or whether admission was for a related symptom, with diagnosis established following inpatient neurological consultation.

### Cost

We have estimated the mean cost of an ALS diagnosis at €3,486 per patient. The basic cost of a standard outpatient diagnostic work-up is €1,414, using the AAN and EFNS criteria.

In our study only six patients underwent unnecessary surgery which is lower than in previous studies. Srinivasan et al. [14] conducted a retrospective chart review of 260 consecutive ALS patients. Fifty five patients (21%) underwent surgery during the five years preceding ALS diagnosis, of whom 34 (61%) underwent surgery for signs and symptoms subsequently deemed attributable to early ALS.

The availability of high quality MRI and detailed neurophysiologic studies in our cohort is likely to have reduced the rate of inappropriate surgical intervention. However our data have indicated that the occurrence of decompressive surgery of the cervical and lumbar spine in patients with ALS is more common than a misdiagnosis of ALS in patients with cervical or lumbar compressive myelopathy. In this context, misdiagnosis of ALS imposes an economic cost and possible surgical complications.

### Savings

There is evidence of widespread waste and inefficiency in the provision of healthcare across both public and private systems. We have shown that a significant portion of this waste arises due to misdiagnosis or delayed diagnosis. Early referral of ALS patients to the MDC could reduce the number of inappropriate consultations and procedures prior to diagnosis, potentially resulting in cost savings. If the diagnostic journey consisted of consultations with a GP and neurologist, as well as lumbar puncture, blood tests, MRI of the brain and spine, and neurophysiology, the minimum cost would be €1,414 per patient. Avoidance of unnecessary consultations and interventions during the diagnostic journey could result in aggregated savings of at least €321,160 (€2,072 per patient). This most likely represents an under-estimate, as in Ireland, the mean length of stay in 2014 for a hospital patient was 7.55 days. For a more representative sample of patients aged 65–74 years undergoing hospital procedures on musculoskeletal or nervous systems the figures are 8.4 and 11.69 days respectively [22]. This suggests that under-reporting of health care utilisation may have occurred.

## Conclusion

A reduction in healthcare spending is possible from improvement in referral pathways to the ALS clinic, and savings may reach around €2,072 per patient. In principle these cost savings could be invested in improved services in the ALS MDC clinic or elsewhere in the health system, and this decision should be guided by standard cost-effectiveness considerations. More appropriate referral to the ALS clinic could also reduce the waiting time for other services, and this could improve timeliness of care and preserve quality of life for ALS patients.

## Supporting information

S1 Appendix Path to MD care. (DOCX)
