# Supplementary material for: The path to specialist multidisciplinary care in amyotrophic lateral sclerosis: A population- based study of consultations, interventions and costs
Source: PLoS One. 2017 Jun 22;12(6):e0179796. doi: 10.1371/journal.pone.0179796 (PMC5480998; doi:10.1371/journal.pone.0179796)
Supplement: S1 Appendix — (DOCX) [file pone.0179796.s001.docx]

# Appendix 1

**Table A1: Predictors of duration from first symptom to diagnosis**

| gap_symp_diag | Coef. | P>\|t\| | 95% Conf. Interval | |
| --- | --- | --- | --- | --- |
| age | 0.071 | 0.402 | -0.096 | 0.237 |
| Private | -2.594 | 0.167 | -6.290 | 1.102 |
| Female | 0.738 | 0.710 | -3.186 | 4.663 |
| Bulbar | -4.246 | 0.040 | -8.294 | -0.198 |
| El_Escorial_Numeric | 1.018 | 0.391 | -1.322 | 3.358 |
| Neur_first_3_binary | -1.297 | 0.504 | -5.127 | 2.532 |
| _cons | 10.883 | 0.087 | -1.590 | 23.356 |

**Table A2: Tobit regression: total direct healthcare costs before diagnosis**

| gap_symp_diag | Coef. | P>\|t\| | 95% Conf. Interval | |
| --- | --- | --- | --- | --- |
| age | 38.973 | 0.254 | -28.318 | 106.265 |
| Private | -6.647 | 0.993 | -1530.923 | 1517.628 |
| Female | -1531.173 | 0.051 | -3071.683 | 9.338 |
| Bulbar | -715.985 | 0.389 | -2355.333 | 923.363 |
| El_Escorial_Numeric | 534.041 | 0.262 | -404.688 | 1472.771 |
| Mitos | 318.903 | 0.620 | -950.333 | 1588.138 |
| Neur_first_3_binary | -2191.913 | 0.006 | -3748.312 | -635.514 |
| _cons | 2091.863 | 0.415 | -2973.137 | 7156.863 |
| /sigma | 3891.988 |  | 3373.106 | 4410.871 |

**Table A3: Tobit regression: direct costs of consultations before diagnosis**

| consults_subtot | Coef. | P>\|t\| | 95% Conf. Interval | |
| --- | --- | --- | --- | --- |
| age | 1.435 | 0.285 | -1.211 | 4.081 |
| Private | -4.206 | 0.890 | -64.305 | 55.893 |
| Female | -19.771 | 0.520 | -80.445 | 40.902 |
| Bulbar | 11.555 | 0.724 | -53.046 | 76.156 |
| El_Escorial_Numeric | 27.409 | 0.144 | -9.547 | 64.364 |
| Mitos | 24.930 | 0.326 | -25.164 | 75.022 |
| Neur_first_3_binary | -67.767 | 0.030 | -128.914 | -6.619 |
| _cons | 333.809 | 0.001 | 135.342 | 532.276 |
| /sigma | 153.712 |  | 133.117 | 174.307 |
